# Testing Online Men’s Groups to Promote Psychological Well-Being and Reduce Despair During the COVID-19 Pandemic

**DOI:** 10.1093/geroni/igab046.2017

**Published:** 2021-12-17

**Authors:** Paul Links, Sisira Sarma, Gordon Flett, Kimberly Wilson, Simon Hatcher, Sylvie Lapierre, David Conn, Marnin Heisel

**Affiliations:** 1 McMaster University, Hamilton, Ontario, Canada; 2 The University of Western Ontario, London, Ontario, Canada; 3 York University, Toronto, Ontario, Canada; 4 University of Guelph, Guelph, Ontario, Canada; 5 University of Ottawa, Ottawa, Ontario, Canada; 6 Universite du Quebec a Trois-Rivieres, Trois-Rivieres, Quebec, Canada; 7 Baycrest; University of Toronto, Toronto, Ontario, Canada; 8 Schulich School of Medicine & Dentistry, The University of Western Ontario, London, Ontario, Canada

## Abstract

Suicide prevention is a healthcare and social justice priority. Older adults have the highest rates of suicide and the highest COVID-19 fatality rates in North America. The combined impacts of social isolation, fear of infection, apathy, and hopelessness could amplify suicide risk among older adults, as appears to have been the case during the 2003 SARS epidemic in Hong Kong. Innovative interventions are thus needed to promote social interaction and reduce risk for suicide in these challenging times. We are currently testing an online version of our Meaning-Centered Men’s Group (MCMG; Heisel et al., 2020), an upstream psychological intervention designed to promote psychological well-being and reduce suicide risk among men struggling with the transition to retirement, in the context of pandemic-related public health restrictions. This presentation will focus on adaptations to MCMG for online delivery, and share participant experiences and findings on positive and negative psychological outcomes.

